# Recent progress in the development of advanced support materials for electrocatalysis

**DOI:** 10.3389/fchem.2023.1304063

**Published:** 2023-11-09

**Authors:** M. Smiljanić, I. Srejić, J. P. Georgijević, A. Maksić, M. Bele, N. Hodnik

**Affiliations:** ^1^ Department of Materials Chemistry, National Institute of Chemistry, Ljubljana, Slovenia; ^2^ Department of Atomic Physics, Institute for Nuclear Sciences Vinča, University of Belgrade, Belgrade, Serbia

**Keywords:** electrocatalysis, fuel cells, electrolyzers, support, metal-support interaction

## Abstract

Electrocatalytic materials are pivotal for clean chemical production and energy conversion in devices like electrolyzers and fuel cells. These materials usually consist of metallic nanoparticles which serve as active reaction sites, and support materials which provide high surface area, conductivity and stability. When designing novel electrocatalytic composites, the focus is often on the metallic sites, however, the significance of the support should not be overlooked. Carbon materials, valued for their conductivity and large surface area, are commonly used as support in benchmark electrocatalysts. However, using alternative support materials instead of carbon can be beneficial in certain cases. In this minireview, we summarize recent advancements and key directions in developing novel supports for electrocatalysis, encompassing both carbon and non-carbon materials.

## 1 Introduction

Electrocatalysis plays an important role in advancing clean energy and chemical production. Renewable-powered water electrolysis produces green hydrogen (Carmo et al., 2013; Lei et al., 2019), while fuel cells convert different fuels (hydrogen, methanol, ethanol, *etc.*) into electricity (Carrette et al., 2001). CO_2_ electrolyzers transform this feedstock into value-added chemicals (Popović et al., 2020; Wang et al., 2023), while ammonia can be produced via nitrogen electro-reduction (Zeng et al., 2020; Lu et al., 2021). The core of these devices are electrocatalysts, typically comprised of metallic nanoparticles (NPs) dispersed on support materials. NPs act as the active sites for reaction enrolling, while supports provide electrical conductivity, a fine distribution of NPs, and stability in (electro)chemical environments.

The focus in the development of novel electrocatalytic materials is primarily on active sites. Strategies for improving intrinsic activity, stability, and selectivity include altering composition (Bing et al., 2010; Mistry et al., 2016; Mukherjee et al., 2022), customizing nanostructures (Erini et al., 2015; L. Pan et al., 2019; Wu et al., 2019) and downsizing catalyst to the level of singe atoms (Li et al., 2019; Speck et al., 2020; Wang et al., 2020). Pt-M alloys (M = Co, Cu, Ni) became benchmark catalysts for fuel cells (Bing et al., 2010; Mistry et al., 2016; Mukherjee et al., 2022). High entropy alloys, formed by mixing five or more metals, create entirely new active sites (Huo et al., 2022). Single-atom electrocatalysts exemplify novel chemistry where isolated atoms yield outstanding performances (Li et al., 2019; Speck et al., 2020; Wang et al., 2020).

Another approach toward novel electrocatalytic composites involves studying the effect of the support on the performance of active sites (Jayabal et al., 2020). Carbon materials are the most common supports in electrocatalysis as they possess unique properties including large surface area and high conductivity (Sharma and Pollet, 2012). Although carbon nanomaterials can serve both as catalysts and catalyst supports (Zhai et al., 2015), they can be enhanced to overcome shortcomings like limited electrochemical stability (Bele et al., 2019; Reier et al., 2014; Pizzutilo et al., 2016). Additionally, the weak interaction between carbon support and NPs may promote certain degradation mechanism such as particle detachment, migration, and coalescence (Campisi et al., 2018). To this end, various directions have been explored, including modification of carbon supports by functionalization, introducing defects and doping with heteroatoms, or developing supports based on transition metal oxides, nitrides, carbides, and organic materials.

This minireview summarizes recent advances in enhancing electrocatalyst performance through support effects. We cover both carbon and non-carbon materials, the two main directions in the field. We explore strategies for improving carbon supports and efforts to design non-carbon materials that combine the benefits of carbon while enhancing active site performance.

## 2 Carbon-based supports

The diverse range of carbon materials available for catalyst substrates highlights their versatility. In this section, the latest research on the most studied and most promising systems will be presented.

### 2.1 Graphene

Graphene is a 2D material consisting of a monolayer of sp^2^ hybridized carbon atoms disposed in a hexagonal packing. Graphene stands out because of large theoretical surface area and easy tenability (Tarcan et al., 2020). Pristine graphene is rarely utilized in electrochemistry, whereas its derivatives like graphene oxide (GO) (Krishnamoorthy et al., 2013) and reduced graphene oxide (rGO) (Erickson et al., 2010) are widely used. The interest in graphene arises from its single-layer structure (Baig et al., 2021) which prevents re-stacking through the formation of a well-organized and interconnected 3D framework. One way to prevent agglomeration is to obtain graphene with more hydroxyl groups at the edges, which can serve also as active sites (Pan et al., 2019).

**FIGURE 1:**
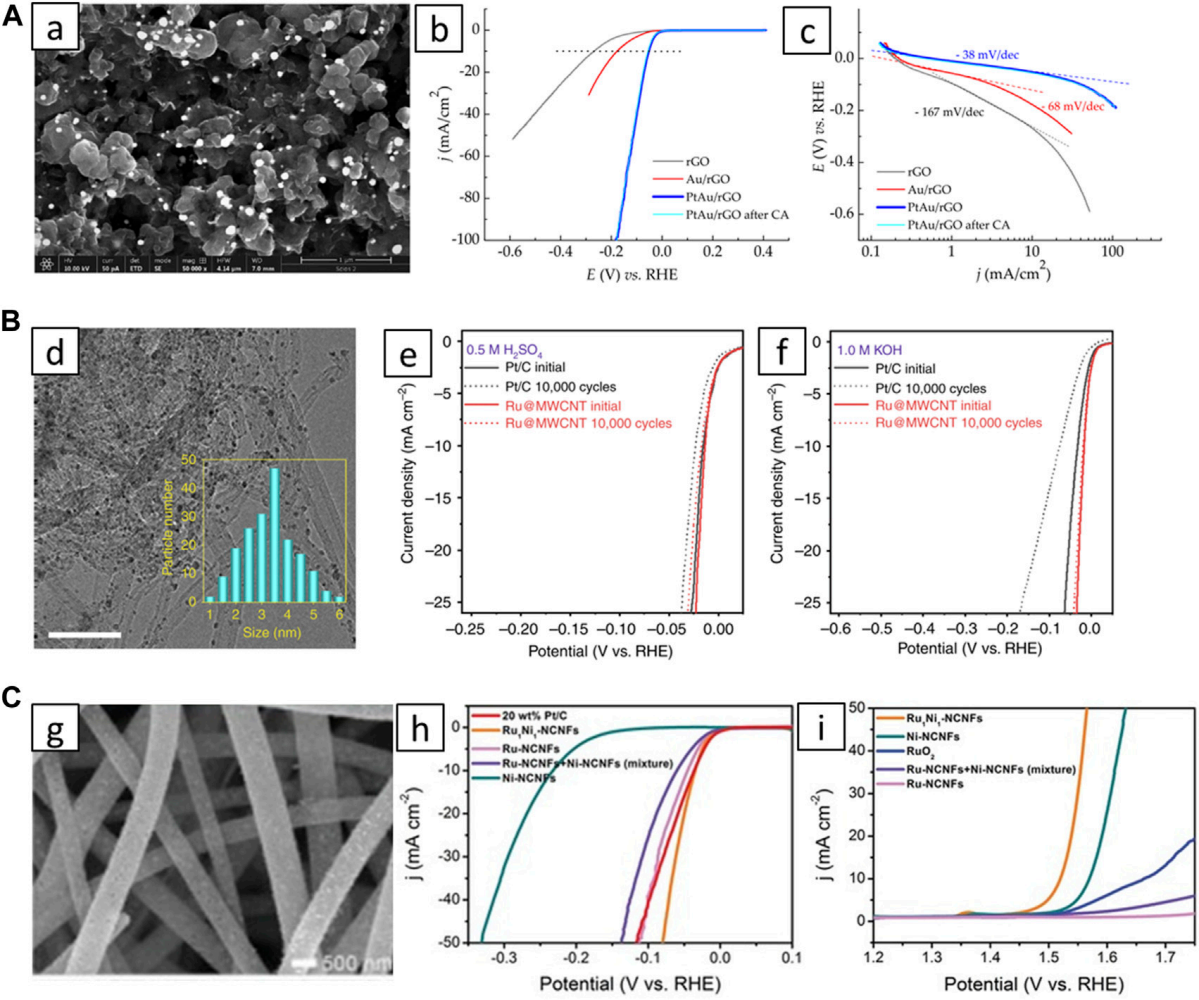
**(A)** (a) SEM image of PtAu/rGO; (b) comparison of HER activities in 0.5 M H_2_SO_4_; (c) corresponding Tafel slopes (Rakočević et al., 2021). **(B)** (d) TEM image of Ru@MWCNTs with particle size distribution; (e) and (f) HER activity and stability compared to Pt/C in different media (Kweon et al., 2020). **(C)** (g) SEM image of Ru_1_Ni_1_-NCNFs; (h) HER and (i) OER polarization curves in 1 M KOH (M. Li et al., 2020).

The interaction between NPs and graphene can be enhanced by heteroatom doping. Xiong et al. (2013) showed that significantly smaller and more homogeneous nanoparticles with enhanced catalytic activity were obtained on N-doped graphene. The introduction of N and B dopants (Y. Sun et al., 2016) can synergistically improve catalyst performance. Pt single-atom catalysts were synthesized using holey N-doped graphene as support and achieved 28 times greater mass activity than Pt/C for hydrogen evolution reaction (HER) (Sun et al., 2022). Our group created advanced PdAu (Rakočević, Srejić, et al., 2021) and PtAu (Rakočević et al., 2021) catalysts on rGO for HER by combining two metals to optimize their electronic structure. Excellent HER activity of PtAu/rGO catalyst can be observed in (Figure 1A). Our recent works revealed that using graphene derivatives as support for Pt-M improves fuel cell catalyst performance. Enhancements were attributed to increased levels of total oxygen, specific oxygen functionalities, sp^2^ carbon, and reduced structural defects (Pavko et al., 2022; 2023).

Combining the porous structure of graphene and doping with heteroatoms can significantly improve catalyst performance (Gao et al., 2023). Karaman (2023) synthesized N,P,S triple-doped 3D graphene architectures with interconnected, hierarchical porous structure, which was decorated with Pd NPs. This catalyst showed improved activity and stability for ethanol oxidation reaction (EOR) compared to Pd@C and Pd@3DG. Ma et al. (2022) developed a one-step strategy for preparing N, P co-doped graphene-supported ultrafine Ru_2_P NPs using GO and deoxyribonucleic acid (DNA) as precursors. In this case, the biomolecule of DNA gives the sites for metal ion adsorption leading to the excellent activity for HER in the whole pH range.

High conductivity of graphene facilitates electron transfer at the interface (Jung et al., 2020) and provides more active sites (Navalon et al., 2017) for electrocatalytic reactions. This is crucial when using poorly conductive materials as catalysts (Li et al., 2017). Bejigo et al. (2023) synthesized Co_3_O_4_/rGO catalysts by recycling spent lithium-ion batteries. The activity of Co_3_O_4_/rGO for oxygen reduction reaction (ORR) is similar to that of commercial Pt/C but with improved stability. Tang et al. (2022) showed that combining the cobalt metaphosphate with 3D graphene support provides a catalyst with promising HER performance.

### 2.2 Carbon nanotubes (CNTs)

CNTs are nanometer-sized cylindrical structures, which can be either single-walled (SW) or multi-walled (MW), depending on the number of concentric layers. Due to their chemical stability, they can be used in different pH environments. High electrical conductivity and large surface area of CNTs enable efficient electron transfer and more active sites (Ortiz-Herrera et al., 2022) making them interesting supports for electrocatalysis.

Wang et al. (2023) developed electrocatalyst for HER by depositing Pt nanoparticles onto activated CNTs. Outstanding HER performance was ascribed to the improved electron conductivity of CNTs and the increased number of exposed active sites. Liu et al. (2023) synthesized three-metallic PtPdRh nanoparticles with CNTs and obtained a highly active catalyst for EOR. Multicomponent catalyst, denoted as Co@CNTs|Ru, showed excellent results for HER in the whole pH range (Chen et al., 2022). The arrangement of Co NPs constrained within nanotubes and a small amount of Ru uniformly deposited on their outer walls led to a redistribution of charges and electron coupling, resulting in excellent HER activity. Wang et al. (2020) reported an HER catalyst containing Co nanoparticles, along with Pt and CNTs. They used dealloying of PtCo/CNT catalyst to reach excellent HER performance. Kweon et al. (2020) uniformly deposited small Ru nanoparticles on MWCNTs modified with–COOH groups, which showed exceptional HER performance in both acidic and alkaline media (Figure 1B). DFT calculations suggest that Ru-C bonds likely serve as the primary active sites for HER. MWCNTs were also utilized for developing low-cost catalysts with MnSe nanostructures for both alkaline oxygen evolution reaction (OER) and ORR (Singh et al., 2022).

Regarding heteroatom doping, nitrogen-doped CNTs (N-CNTs) are the most widely studied. For example, excellent catalysis was obtained on N-CNTs supporting Pt-Ru for methanol oxidation reaction (MOR) (Forootan Fard et al., 2020). Similarly, Cu-Fe oxide alloy nanoparticles supported on N-CNTs exhibited promising activities for HER and ORR (Liu et al., 2022). The B,N co-doped CNTs with highly dispersed Ru nanoclusters showed high activity for water splitting. Simultaneous doping with B and N significantly reduces the adsorption energy of the hydrogen intermediate at Ru sites, which enhances the HER kinetics (Fan et al., 2022). S-doped (Tavakol et al., 2020) or P-doped (Song et al., 2022) CNTs have also shown potential in improving HER kinetics.

### 2.3 Other carbon-based supports

In addition to graphene and CNTs, various other carbon-based materials have potential as catalyst substrates. Carbon nanofibers (CNFs) are similar to CNTs in structure but have a more disordered arrangement. Wang et al. (2017) synthesized well-dispersed Pt-Cu nanoparticles enclosed in CNFs as a highly effective catalyst for HER. The outstanding performance originated from synergistic interaction between Pt and Cu, the uniform distribution of the alloy nanoparticles, and the use of CNFs with 3D architectures. Hodnik et al. reported a facile and scalable synthesis of 3D-structured electrodes composed of Pt NPs deposited on graphitized CNFs which were used as catalysts for ORR and MOR (Hodnik et al., 2020). RuNi nanoparticles grafted in nitrogen-doped CNFs also showed very good activity for water-splitting reactions (Figure 1C) (Li et al., 2020).

Carbon aerogels (CA) are 3D structures that can be organic-based, graphitic materials-based, or biomass-based (Lee and Park, 2020). The aerogel’s porosity affects the distribution and size of the NPs and inhibits their aggregation (Peles-Strahl et al., 2023). Li et al. synthesized a highly stable non-precious metal catalyst with template-assisted few-layer graphene aerogel as support with high activities for OER and ORR (Li et al., 2023). Hou et al. (2023) developed a Ni-WC/CA anode for MOR, using the highly conductive bacterial cellulose-derived carbon as the substrate.

Another strategy to create catalysts with excellent corrosion resistance involves the combination of fullerene C60 and platinum. The attachment of Pt clusters onto two-dimensional fullerene nanosheets (Chen et al., 2023), as well as confining single Pt atoms using two fullerene molecules were achieved (Zhang et al., 2023). In both cases, the enhanced catalytic effect originated from the varied bonding characteristics of Pt-sites at the Pt/C60 interface. Ruthenium nanoparticles and singe atoms were grafted into a 3D crystalline fullerene network, providing an outstanding catalyst for alkaline HER (T. Luo et al., 2023).

## 3 Non-carbon-based supports

Despite the mentioned advantages and feasibility of carbon supports, there are cases where alternative materials are necessary. In this section, we will explore recent advancements in the design of non-carbon supports for electrocatalysis.

### 3.1 Transition metal oxides-based supports

Transition metal oxides (TMOs) are attractive in electrocatalysis due to their (electro)chemical stability and ability to trigger metal-support interaction (MSI) with active sites (Tauster et al., 1978; Pan et al., 2017; Luo et al., 2022). However, TMOs often suffer from limited conductivity. For example, TiO_2_, a cost-effective, low-toxicity semiconductor with excellent corrosion resistance. To address insufficient conductivity, Kwon et al. developed a TiO_2_/C composite by covering carbon with a sub-nanometer-thick TiO_2_ layer, which enhanced the durability of carbon support and the performance of supported Pt for ORR through MSI (Shi et al., 2021). MSI weakens the interaction of Pt with intermediate species, which results in more active sites available for O_2_ adsorption. Gasteiger’s group achieved a different effect by partially reducing a TiO_2-y_ layer on carbon and decorating it with Pt nanoparticles using atomic layer deposition (Geppert et al., 2020). This led to the encapsulation of Pt particles with a TiO_2-y_ coating, enabling selective catalysis of hydrogen reactions while inhibiting ORR, CO oxidation, and Pt oxidation. Such selectivity is crucial for fuel cells’ lifetime, as it prevents catalyst deactivation due to the enrolling of the undesired ORR at Pt-based anodes (Jung et al., 2020). A similar encapsulation effect was demonstrated for Ru/TiO_x_/C composites (Stühmeier et al., 2022). In summary, blending TiO_2_ with carbon is a viable strategy to enhance its conductivity.

Another way to increase TiO_2_ conductivity is transition metal doping. For instance, incorporating W into TiO_2_ generates a highly conductive composite with a high surface area (Pham et al., 2021). Pt supported on W-doped TiO_2_ (Pt/W-TiO_2_) exhibited significantly improved ORR activity compared to undoped Pt/TiO_2_ and surpassed the durability of Pt/C (T. M. Pham et al., 2023). A similar effect was shown for Pt supported on Ta-doped TiO_2_ (Liu et al., 2014; Anwar et al., 2017) and Nb-doped TiO_2_ (Liu et al., 2014). Analogously, IrO_2_ and IrRuO_x_ nanoparticles loaded on Nb-doped TiO_2_ nanotubes exhibited high activity for OER (Genova-Koleva et al., 2019).

**FIGURE 2:**
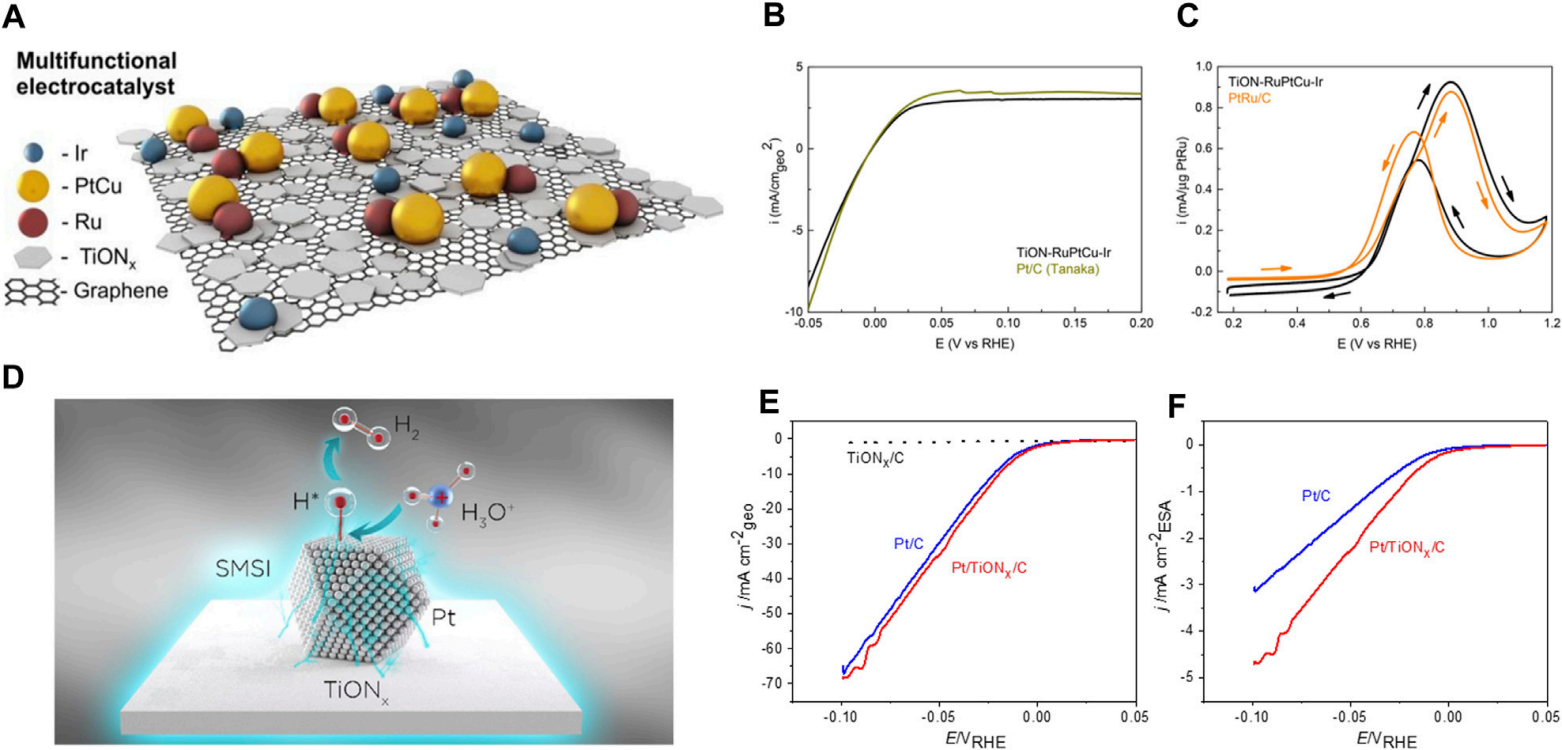
**(A)** Scheme of multifunctional electrocatalyst comprised of Ir, PtCu and Ru nanoparticles dispersed over TiON-graphene support; **(B)** comparison of HER/HOR activity of TiON-RuPtCu-Ir catalyst with Pt/C benchmark; **(C)** comparison of MOR performance of TiON-RuPtCu-Ir catalyst with PtRu/C bechmark; **(D)** scheme of MSI between Pt and TiON; **(E)** comparison of HER activities of TiON/C, Pt/TiON/C and Pt/C; **(F)** intrinsic HER activities of Pt/C and Pt/TiON/C obtained by taking into account Pt electrochemical surface area. Reprinted with permission from Moriau et al. (2019); Smiljanić et al. (2022). Further requests regarding reproduction of the used materials should be directed to the ACS.

Our group has developed a way to increase the conductivity of TiO_2_ using partial nitridation to form titanium-oxynitride (TiON). Initial work by Bele et al. showed that TiON is a conductive material capable of anchoring small-sized Ir nanoparticles (Bele et al., 2019). The OER activity and stability of the Ir/TiON composite surpassed the performance of Ir-benchmarks. Further developments included mixing TiON with carbon to increase its surface area. Ir/TiON/C composite with nearly three times higher OER mass activity than Ir-black was reported by Loncar et al. (2020). Ir nanoparticles immobilized on nanotubular TiON exhibited exceptional OER performance due to MSI. The presence of nitrogen was shown to be crucial for Ir anchoring and nanoparticle stability (Bele et al., 2020). Another work reported that the presence of Ir stabilized TiON support against oxidation during electrochemical treatments (Koderman Podborsek et al., 2022). We have also explored the effect of TiON on other catalysts and reactions beyond Ir and OER. Graphene covered with TiON was decorated with PtCu, Ir and Ru nanoparticles (Moriau et al., 2019). This composite (scheme given in Figure 2A) exhibited high catalytic performance for various reactions occurring in fuel cells and electrolyzers (Figures 2B, C). More recently, we have shown that Pt/TiON outperforms Pt/C as a HER catalyst due to the dual effect of MSI (Figures 2D–F): i) adjustment of H adsorption energetics; ii) more effective anchoring of Pt particles with respect to carbon (Smiljanić et al., 2022).

Tin-oxide (SnO_2_) is another interesting material in this context. Sasaki et al. demonstrated that Pt/SnO_2_ composite is more stable than Pt/C in fuel cell operations and that doping of SnO_2_ with hypervalent anions (Nb^5+^, Sb^5+^, Al^3+^) can increase its conductivity (Takasaki et al., 2011). The approach of doping antimony into tin-oxide (ATO) and decoration with Pt was adopted for obtaining better-performing catalysts for ORR (Hussain et al., 2019; He et al., 2020), and electro-oxidation of methanol and ethanol (Lee et al., 2008). In terms of OER, ATO is considered state of the art support for iridium nanocatalysts (Puthiyapura et al., 2014). Many works reported impressive OER catalysts prepared by supporting IrO_2_ on various types of ATO (Tong et al., 2017; Han et al., 2020; Moriau et al., 2022; Khan et al., 2023). The advantages of SnO_2_ over carbon include higher durability, an oxophilic effect beneficial for CO tolerance of supported nanoparticles, and the ability to induce MSI.

Tungsten-oxide (WO_3_) also found application in electrocatalysis research. Recent findings reveal that Pt/WO_3_ has remarkable HER activity, attributed to electron transfer from the support to Pt sites, leading to the adjustment of the free energy of H adsorption (Fan et al., 2023). Conversely, Kim et al. reported a unique effect of WO_3_ support, known as a metal-insulator transition (Jung et al., 2020). When exposed to hydrogen, WO_3_ transforms into a metallic conductor (H_x_WO_3-x_), enabling supported Pt to catalyze HOR. In contrast, H_x_WO_3-x_ reverts to insulating WO_3_ in an oxygen atmosphere and suppresses ORR. Such selective catalysis of Pt-materials is beneficial for the fuel cell lifespan.

### 3.2 Other non-carbon-based supports

Many other materials gained attention in electrocatalysis, including nitrides, carbides, 2D-materials, organic matrices, *etc.* Transition metal nitrides are known for high conductivity which makes them interesting for electrocatalysis. For instance, Ir nanoparticles loaded onto TiN exhibited improved OER performance due to electronic interactions between TiN and Ir (G. Li et al., 2018). A recent study also reported electron transfer between Ru nanorods anchored on TiN, contributing to the excellent HER activity of Ru/TiN composite (Yang et al., 2022). However, the instability of TiN is a concern for its electrocatalytic applications (Avasarala and Haldar, 2011). To address this, DiSalvo et al. prepared a ternary Ti_0.5_Nb_0.5_N nitride as a support for Pt, which exhibited better ORR performance than Pt/C (Cui et al., 2013). Similar benefits were observed with various transition metal carbides. Sun and others synthesized a RuO_2_-WC composite with exceptional activity for water-splitting, attributed to the regulation of Ru electronic structures via MSI (Sun et al., 2022). Ru singe atoms supported on nitrogen-doped molybdenum carbide displayed excellent HER performance due to the synergistic effect between support and active Ru sites (Yu et al., 2020).

Recently, a group of 2D layered materials called MXenes gained prominence in electrocatalysis (Liu et al., 2020; Bai and Guan, 2022). For instance, Pt nanoclusters deposited on Ti_3_C2T_x_ MXene exhibited higher HER activity than Pt/C (Jian et al., 2021). Sun’s team reported MXene catalysts with low Pt loading and impressive HER performance, which was linked with the influence of surface terminals (OH and O) in MXene on the electronic state of Pt (Yuan et al., 2019).

Our group recently explored tris(aza)pentacene (TAP) as a support for Pt nanoparticles (Vélez Santa et al., 2021; Smiljanić, Bele, et al., 2022). TAP belongs to the family of polyheterocycles, which are organic compounds interesting in various fields of chemistry and material science due to their electronic, optical, and conductive properties. In aqueous media, TAP features tunable conductivity due to reversible protonation/deprotonation. When protonated at lower potentials (<0.5 V_RHE_), the conductivity of TAP increases, allowing supported Pt to run HOR/HER, while at higher potentials limited TAP conductivity prevents Pt/TAP from running ORR (Vélez Santa et al., 2021). Additionally, this feature of TAP reduces Pt dissolution during rapid potential spikes encountered during device switching on/off, which is otherwise very harmful for Pt/C benchmarks (Smiljanić, Bele, et al., 2022).

## 4 Summary and outlook

This mini-review provides a concise overview of recent advancements in designing support materials for electrocatalysis. High surface area carbon-based materials have shown their versatility in electrocatalysis but also have room for further improvements. The most important challenge is in optimizing the activity of supported nanoparticles and enhancing the carbon corrosion resistance to prevent degradation of the catalytic composites. High-temperature treatments that lead to graphitization offer a path to more stable materials, but this comes with the trade-off of potentially reducing catalytic activity by removing defects and heteroatoms. Strategies for manufacturing advanced carbon-based materials will be the focus of future research. Despite all advantages, in some cases, however, it is necessary to replace carbon with some alternative supports. After addressing their low conductivity, TMOs offer several key advantages over carbon in tackling specific challenges in energy conversion devices. Their corrosion resistance enables the design of supported nanocatalysts for OER, which significantly improves the utilization of precious and scarce metals such as Ir and Ru. The ability to induce MSI and to improve the activity and durability of supported active sites can be used to create advanced electrocatalysts for a range of reactions. Materials such as ATO and TiON are certainly TMO derivates with interesting features as electrocatalytic supports for different reactions. Further work is needed to additionally understand the MSI provided by these supports for each particular case (i.e., each combination of metal and reaction), which will allow more straightforward tailoring of the advanced electrocatalytic composites. Similar is valid for nitrides, carbides, MXenes, and organic matrices, which can be used for reactions in both fuel cells and electrolyzers.

Overall, we anticipate that carbon materials will remain a key focus in electrocatalysis, particularly in cases when they provide sufficient durability for stable catalyst operation. Non-carbon supports are foreseen to play a role when a specific issue is to be addressed (such as carbon corrosion) and when additional enhancement of the performance of active sites via MSI is desired. While TMOs, nitrides, and carbides are already well-known as supports for electrocatalysis, the research on MXenes and a few other types of 2D materials (phosphorenes, boridenes, *etc.*) is in its early stages, offering ample opportunities for the synthesis of advanced electrocatalytic composites. In the case of non-carbon-based materials, the choice of support can be guided by the specific demand in front of the catalyst.
